# Maternal Serum Angiogenic Factor sFlt-1 to PlGF Ratio in Preeclampsia: A Useful Marker for Differential Diagnosis and Prognosis Evaluation in Chinese Women

**DOI:** 10.1155/2019/6270187

**Published:** 2019-07-16

**Authors:** Wei-zhen Lou, Fang Jiang, Jing Hu, Xiao-xu Chen, Ying-na Song, Xi-ya Zhou, Jun-tao Liu, Xu-ming Bian, Jin-song Gao

**Affiliations:** Department of Obstetrics & Gynecology, Peking Union Medical College Hospital, Peking Union Medical College, Chinese Academy of Medical Sciences, Beijing 100730, China

## Abstract

The ratio of soluble fms-like tyrosine kinase-1 to placental growth factor (sFlt-1/PlGF) is elevated and proved to be useful in preeclampsia (PE) diagnosis. Its value in differential diagnosis with other pregnancy complications and prediction of pregnancy duration has yet to be clarified in Chinese population. We retrospectively analyzed 118 singleton pregnancies with suspected or diagnosed PE at the Peking Union Medical College Hospital (PUMCH) in China. Among these, 62 pregnancies were diagnosed as PE (48 early onsets and 14 late onsets, with 39 and 5 severe PE, respectively), 12 gestational hypertension (GH), 15 chronic hypertension (chrHTN), 16 autoimmune diseases, and 13 pregnancies with uncomplicated proteinuria. And 76 normal pregnancies were included as control. The results showed (1) the sFlt-1/PlGF ratio in early onset PE subgroup was significantly higher than that in GH, chrHTN, and control groups; the sFlt-1/PlGF ratio in late onset PE subgroup was significantly higher than that in chrHTN and control groups, but similar as GH group; the sFlt-1/PlGF ratio was similar among GH, chrHTN, and control groups. (2) The sFlt-1/PlGF ratio was significantly increased in the PE group compared with autoimmune disease and uncomplicated proteinuria pregnancies. (3) By ROC curve analysis, the cutoff value of the sFlt-1/PlGF ratio was less than 21.5 to rule out PE and higher than 97.2 to confirm the diagnosis of PE. (4) The sFlt-1/PlGF ratio was higher in PE pregnancies delivering within 7 days than those more than 7 days, either in early onset PE or severe PE. In conclusion, we show that maternal sFlt-1/PlGF ratio is an efficient biomarker in the diagnosis and differential diagnosis of PE. This ratio can be used to predict the timing of delivery for PE pregnancies.

## 1. Introduction

Preeclampsia (PE) affects 5~8% of pregnancies worldwide and remains as a major cause of morbidity and mortality in both the mother and the fetus worldwide [1–4]. PE is of special relevance in the developing countries, where the maternal mortality is ∼15% compared with 0∼1.8% in the developed countries [5]. In China, diagnosis of PE is mostly based on limited clinical signs and unspecific laboratory findings, which are compromised to make early diagnosis and proper management. Sometimes, it is difficult to differentiate PE from predisposing conditions, such as chronic hypertension, CKD (chronic kidney disease), and autoimmune diseases like systemic lupus erythematosus which are also the leading risk factors for PE. Meanwhile, the outcome of PE is hard to predict due to diverse disease progression in individuals [6]. It is valuable to find out a specific marker that can improve diagnostic accuracy, help determine the timing of delivery, and thus improve the outcome of PE.

In the last decade, our understanding of the pathogenesis underlying PE has progressively advanced and it is generally appreciated that early and late onset PE have different pathophysiologies [7–11]. Early onset PE, also referred to as placental PE, arises from a placenta that is under hypoxic conditions with oxidative stress [12], while late onset PE, also called maternal PE, arises from the interaction between a normal placenta and a maternal constitution that is susceptible to, or suffers from, metabolic and vascular diseases, as with long-term hypertension or diabetes [13, 14]. Mixed presentations, combining maternal and placental contributions, are common [15]. Placental PE is characterized by defective deep trophoblastic invasion and impaired maternal spiral artery remodeling in the first half of pregnancy, leading to inadequate placental perfusion in the second half. The state of placental insufficiency triggers an imbalance in the placental release of angiogenesis regulatory factors to the maternal circulation, characterized by decreased concentrations of proangiogenic factors such as placental growth factor (PlGF) and elevated concentrations of prohypertensive and antiangiogenic factors such as soluble fms-like tyrosine kinase-1 (sFlt-1) [16, 17]. PlGF, a member of the VEGF family, can promote placenta angiogenesis, increase vascular permeability, and enhance trophoblast cell activity. Soluble fms-like tyrosine kinase-1 (sFlt-1) can decrease the serum concentration of PlGF, inhibit the biological function of PlGF, and impair the permeability and integrity of vascular wall, leading to angiogenesis disorders, edema, urine protein, and hemoconcentration [18, 19]. Increasing evidences showed that there were elevated serum concentration of sFlt-1 and declined serum concentration of PlGF in PE patients and that the degree of elevated or declined level was correlated with the severity of PE [20]. Because of this, sFlt-1 and PlGF were considered as the most promising serological indicators for PE diagnosis. Particularly, the sFlt-1/PlGF ratio could better reflect the antiangiogenic activity and could be used to predict the occurrence and prognosis of PE [20–22]. However, cutoff values and predictive efficacy varied largely across study countries, suggesting it may not necessarily be similar in different ethnic and geographical populations [23–27]. There were few such studies in Chinese population, and its value in differential diagnosis with other similar pregnancy complications and prediction of pregnancy duration has yet to be clarified.

The objectives of this study are, firstly, to compare the sFlt-1 to the PlGF ratio among PE, other pregnancy complications, and normal controls; secondly, to determine a possible cutoff value aiding to make PE diagnosis; and lastly, to clarify the value of this ratio in the prediction of pregnancy duration in Chinese population.

## 2. Materials and Methods

### 2.1. Study Population

A hundred and eighteen singleton pregnancies with suspected or diagnosed PE after twenty weeks of gestation were enrolled in our study from Feb 2013 to Nov 2016, who underwent regular maternity examinations in our hospital. Maternal blood was collected and serum was frozen and stored at −80°C for analysis. They were closely monitored and treated according to clinical routines. Final diagnosis was confirmed after birth for each patient. The study was approved by the Ethic Committee of Peking Union Medical College Hospital. Written informed consents were obtained for all the women agreeing to use their banked serum for research purposes.

The diagnosis of gestational hypertensive disorders was defined and classified according to the ACOG definition [28]. PE was defined as hypertension (blood pressure ≥140 mmHg systolic and/or ≥90 mmHg diastolic) on two separate occasions at least 4 hours apart at or after 20 weeks of gestation accompanied by proteinuria (24 hour urine protein ≥0.3 g, spot urine protein/creatinine ratio (PCr) ≥0.3, or spot urine dipstick ≥“2+”) or evidence of end-organ dysfunction (i.e., thrombocytopenia, severe headache, renal insufficiency, impaired liver function, and heart and lung dysfunction). Chronic hypertension (chrHTN) was defined as hypertension that occurred in <20 weeks of gestation and lasted ≥12 weeks after delivery without proteinuria. Gestational hypertension (GH) was defined as hypertension that occurred after 20 weeks of gestation without proteinuria or end-organ dysfunction.

Severe PE refers to PE that reaches any of the following criteria [29–31]: (1) persisting elevated severe hypertension: blood pressure ≥160 mmHg systolic and/or ≥110 mmHg diastolic; (2) neurological complications (examples include but not restricted to persistent severe headaches and visual disturbances); (3) persistent right upper quadrant or epigastric abdominal pain; (4) elevated transaminases (e.g., ALT or AST); (5) renal function insufficiency: proteinuria >2.0 g/L, oliguria (24-hour urine less than 400 mL or urine per hour less than 17 mL), or serum creatinine >106 *μ*mol/L; (6) hypoproteinemia accompanied by ascites, hydrothorax, or hydropericardium; (7) hematological complications (thrombocytopenia-platelet count below 100,000/*μ*L, hemolysis manifested by anemia, and elevated lactate dehydrogenase level and jaundice); (8) heart failure; (9) pulmonary edema; and (10) uteroplacental dysfunction (such as fetal growth restriction, oligohydramnios, placenta abruption, or stillbirth).

Delivery plan was based upon maternal and fetal-placental conditions [32]: (1) GH and PE that do not deteriorate to severe PE were managed conservatively until 37 gestational weeks; (2) offer birth to women with severe PE after 34 weeks when their blood pressure has been controlled and a course of corticosteroids has been completed; (3) offer birth to women with severe PE before 34 weeks (with a course of corticosteroids if possible) if severe hypertension develops refractory to treatment or maternal or fetal's adverse complications develops.

PE patients were classified as early onset (<34 weeks of gestation) or late onset (≥34 weeks of gestation) at the time when the diagnosis of PE was made. Remaining duration of pregnancy refers to the period from enrollment to delivery.

### 2.2. sFlt-1 and PlGF Assays

Automated assays for sFlt-1 and PlGF (with both levels measured in picograms per milliliter) were performed using the fully automated Elecsys assays on an electrochemiluminescence immunoassay platform (cobas E 601 immunoassay analyzers, Roche Diagnostics) [33]. The interassay coefficient of variance for sFlt-1 and PlGF immunoassays ranged from 2.6 to 3.0% and 2.0 to 2.4%, respectively. Test results were blinded to both patients and their physicians.

### 2.3. Statistical Analysis

The sFlt-1/PlGF ratio (value of sFlt-1 measured in pg/mL, divided by the value of PlGF measured in pg/mL) was used as a measure of circulating angiogenic imbalance. Box-whisker plots were generated to compare the sFlt-1/PlGF ratio between groups. Baseline characteristics and the sFlt-1/PlGF ratio of patient groups were compared using the *t*-test and the chi-squared test which were appropriate. Independent *t*-test was used for normally distributed continuous variables and the chi-squared test (*χ*^2^) for categorical variables. Variance analysis was applied to examine differences in the main predictors of interests. When the variance was homogenous, the Bonferroni (B) test was performed; otherwise, Tamhane's T2 (M) test was performed. We also used the receiver operating characteristic (ROC) analysis to determine the cutoff value of the sFlt-1/PlGF ratio in the diagnosis of PE. The sensitivity, specificity, and positive and negative predictive values of the cutoff value were calculated.

All *P* values reported were 2-tailed. The *P* values of < 0.05 were considered statistically significant. All statistical analyses were performed with the use of Statistical Package for Social Sciences (SPSS, version 22.0).

## 3. Results

### 3.1. Clinical Features of the Study Population

A total of 118 singleton pregnancies included 62 PE (48 early onsets and 14 late onsets, with 39 and 5 severe PE, respectively), 12 GH, 15 chrHTN, 16 CTD (13 systemic lupus erythematosus, one Sjogren's syndrome, one undifferentiated connective tissue disease, and one antiphospholipid syndrome), and 13 uncomplicated proteinuria. For the control group, 76 normal singleton pregnancies were included. There were no significant differences in maternal age, parity, and gestational week of enrollment among groups. Women with PE had a lower neonate birth weight (1624 ± 704 g) and an earlier gestational week of delivery (32 ± 4 w) compared with healthy control (3198 ± 388 g, 39 ± 2 w) or women with GH, chrHTN, CTD, or uncomplicated proteinuria (*P* < 0.001) (Table 1).

### 3.2. sFlt-1/PlGF Ratio in Different Groups

The sFlt-1/PlGF ratio was higher in PE patients (257.7 ± 44.7) than in normal control (4.1 ± 2.5) or other groups (GH, chrHTN, CTD, and uncomplicated proteinuria patients, with mean level ranged from 8.9 to 38.6, *P* < 0.001). There was no difference between other groups and healthy control (Table 1).

**Figure 1:**
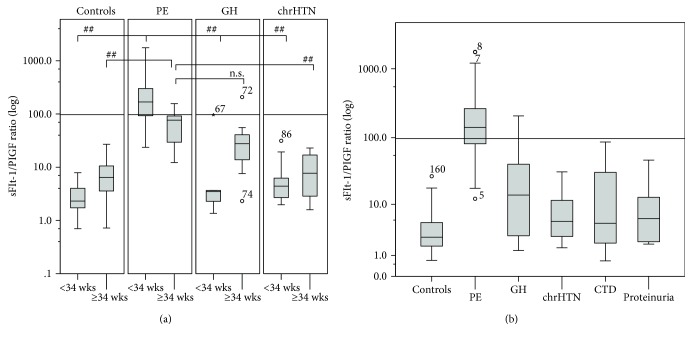
sFlt-1/PlGF ratio in PE, GH, chrHTN, CTD, proteinuria, and controls. Box-whisker plots displayed the sFlt-1/PlGF ratio in patients with PE, GH, chrHTN, pregnancies with CTD or proteinuria, and controls. Boxes indicate interquartile range; whisker indicates range; error bars indicate median. ○ means mild outliers. ^∗^ means extreme outliers. The cutoff of 97.2 was drawn into the graph as a solid line. (a) The logarithmic distribution of the sFlt-1/PlGF ratio in patients with PE/GH/chrHTN/controls. The average Flt-1/PlGF ratio was significantly higher in PE <34 weeks (*n* = 48) than in GH (*n* = 5), chrHTN (*n* = 10), or controls (*n* = 59) <34 weeks (all *P* < 0.001). PE ≥34 weeks (*n* = 14) was compared with chrHTN (*n* = 5) and controls (*n* = 17) ≥34 weeks (all *P* = 0.001), though the difference did not reach statistical significance between PE and GH ≥34 weeks (*n* = 7) (*P* = 0.984). (b) The logarithmic distribution of the sFlt-1/PlGF ratio in patients with PE, GH, chrHTN, pregnancies with CTD or proteinuria, and controls. The sFlt-1/PlGF ratio was higher in PE than in GH, chrHTN, CTD (*n* = 16), proteinuria (*n* = 13), and controls (all *P* < 0.001), while no significant differences between any other two groups.

Early onset PE had higher sFlt-1/PlGF ratio compared with late onset PE (311.9 ± 55.4 vs. 71.7 ± 11.7, *P* < 0.001). In the window of <34 weeks of gestation (early onset), the average Flt-1/PlGF ratio was significantly higher in PE patients than in GH, chrHTN, or healthy control (*P* < 0.001). In the window of ≥34 weeks of gestation, the average ratio in PE is also increased (*P* = 0.001) but the difference between PE and GH groups did not reach statistical significance (71.7 ± 11.7 vs. 50.8 ± 26.9, *P* = 0.984) (Table 2). Patients with GH or chrHTN had a slightly higher sFlt-1/PlGF ratio either before or after 34 weeks of gestation compared with the control group, but this difference did not reach statistical significance (Table 2 and Figure 1).

Severe PE patients (44/62, 314.2 ± 60.0) had higher sFlt-1/PlGF ratio than the mild ones (18/62, 119.5 ± 29.2, *P* = 0.005). At <34 weeks, the sFlt-1/PlGF ratio in severe PE patients was higher than mild patients, but the difference was not significant (344.7 ± 66.1 vs. 169.9 ± 53.0, *P* = 0.221). After 34 weeks, the average ratios of severe and mild PE were similar (76.1 ± 24.2 vs. 69.2 ± 13.5, *P* = 0.791).

### 3.3. Cutoff Value of sFlt-1/PlGF Ratio in the Diagnosis of PE

**Figure 2:**
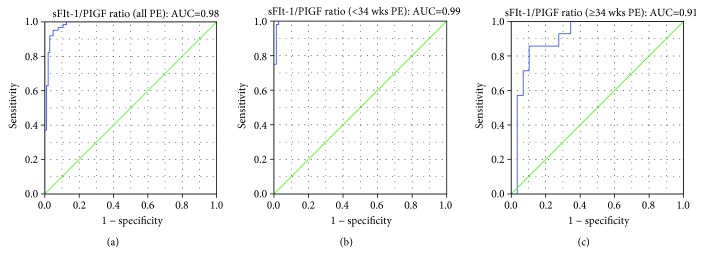
ROC curves for PE diagnosis. Receiver operating characteristic (ROC) curves of the sFlt-1/PlGF ratio were constructed in all PE (AUC = 0.98, 95% CI, 0.969-1.000), early onset PE (AUC = 0.99, 95% CI, 0.989-1.000), and late onset PE (AUC = 0.91, 95% CI, 0.822-1.000). When cutoff of 21.5 was used, the NPV for the diagnosis of PE was 97.9%, 100%, and 91.7% for all, early onset, and late onset PE. When cutoff of 97.2 was used, the PPV for the diagnosis of PE was 97.5%, 100%, and 75.0% for all, early onset, and late onset PE.

In order to determine the performance of the sFlt-1/PlGF ratio in clinical settings, ROC curves were constructed. The sFlt-1/PlGF ratio had an AUC of 0.98 (95% CI, 0.969-1.000), 0.99 (95% CI, 0.989-1.000), and 0.91 (95% CI, 0.822-1.000) for all PE, early onset PE (<34 weeks), and late onset PE (≥34 weeks), respectively (Figure 2).

The sFlt-1/PlGF ratio of 21.5 had the best performance to rule out PE before 34 weeks of gestation, with the NPV of 97.9%, 100%, and 91.7% for all, early onset, and late onset PE. Meanwhile, the value of 97.2 had the best performance to confirm the diagnosis of PE before 34 weeks, with the PPV of 97.5%, 100%, and 75.0% for all, early onset, and late onset PE. This result confirmed the potential clinical usage of the sFlt-1/PlGF ratio for the diagnosis of PE especially before 34 weeks of gestation.

If we utilize the reported cutoff value [6] in our Chinese cohort, using 85 for early onset PE and 111 for late onset PE to confirm diagnosis, the PPV value will be 95.7%, 97.4%, 85.7% and 97.4%, 100.0%, 75.0% accordingly. These results suggest that the reported value is also feasible in our population.

### 3.4. sFlt-1/PlGF Ratio and Remaining Duration of Pregnancy

**Figure 3:**
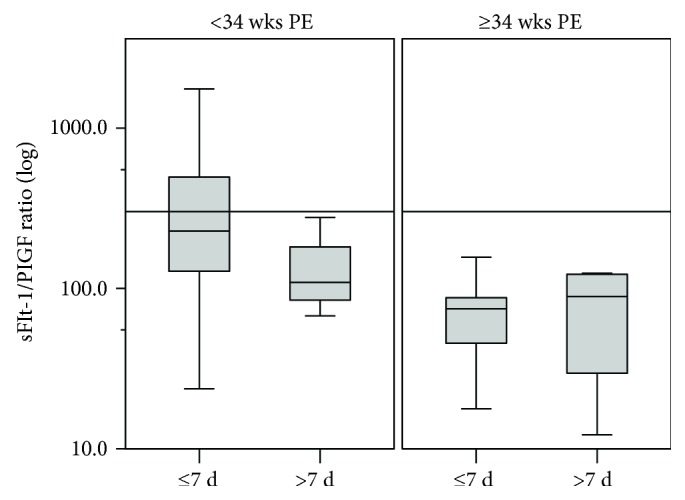
Correlation between sFlt-1/PlGF ratio and time of delivery in PE patients. Box-whisker plots displayed the logarithmic distribution of the sFlt-1/PlGF ratio in two subgroups of PE patients: delivered within 7 d (*n* = 45) and longer than 7 d (*n* = 17) from the time enrolled in the hospital. Boxes indicate interquartile range; whisker indicates range; error bars indicate median. ○ means mild outliers. ^∗^ means extreme outliers. The sFlt-1/PlGF ratio of 301.0 was drawn into the graph as a solid line. The sFlt-1/PlGF ratio in the subgroup delivered within 7 d was significantly higher than the subgroup delivered longer than 7 d (*P* = 0.004).

As time to delivery is an essential indicator of disease severity, we divided the 62 PE patients into two subgroups: delivered within 7 days (45/62) or longer than 7 days (17/62) from the time of enrollment. The sFlt-1/PlGF ratio in the subgroup delivered within 7 d was significantly higher than the subgroup delivered longer than 7 d (309.4 ± 59.6 vs. 120.9 ± 18.0, *P* = 0.004). We also compared the differences between the two subgroups in early onset (48/62), severe (44/62), or early onset severe PE patients (39/62). The sFlt-1/PlGF ratios in subgroups delivered within 7 d were also significantly higher than that in subgroups longer than 7 d whether it is early onset PE (369.3 ± 71.1 vs. 139.8 ± 21.8, *P* = 0.004), severe PE (372.4 ± 74.7 vs. 116.4 ± 25.1, *P* = 0.002), or early onset severe PE (409.8 ± 82.3 vs. 128.0 ± 24.9, *P* = 0.002). There were no significant differences between the two subgroups in late onset PE (69.6 ± 13.9 vs. 75.4 ± 23.3, *P* = 0.822) or mild PE (114.6 ± 45.6 vs. 127.2 ± 27.3, *P* = 0.841) (Figure 3).

The third quartile (Q3) of the sFlt-1/PlGF ratio was 301.0 in early onset PE patients. Of the 48 early onset preeclampsia patients, 12 patients (12/48) had sFlt-1/PlGF ratio greater than Q3, all of them delivered within 7 days. In the 36 patients (36/48) whose Flt-1/PlGF ratio were below Q3, 24 (24/36, 66.7%) were delivered within 7 days (100% vs. 66.7%, *P* = 0.021). One early onset mild PE patient who had a Flt-1/PlGF ratio greater than Q3 was delivered 4 days after recruitment. None of the late onset patient had a ratio higher than 301.0.

## 4. Discussion

### 4.1. The sFlt-1/PlGF Ratio Is Useful in the Diagnosis and Differential Diagnosis of PE

The diagnosis of PE is mainly based on clinical manifestations and unspecific biochemical makers. Some patients with certain complications like chrHTN and CTD are doomed to progress into PE while others will not. These patients may have similar symptoms with PE while the management and prognosis are much different. Therefore, it is essential to make correct diagnosis. However, sometimes, it is difficult to make correct differential diagnosis merely based on current diagnosis criteria. Recently, some researchers reported the use of the sFlt-1/PlGF ratio in the prediction and diagnosis of PE [21, 23, 33–37]. We proved that the sFlt-1/PlGF ratio is also valuable in the diagnosis and differential diagnosis of PE in Chinese population. The sFlt-1/PlGF ratio is markedly increased in PE patient compared with GH, chrHTN, or other medical complications like autoimmune disease and uncomplicated proteinuria. And it is more favorable in diagnosing PE before 34 weeks of gestation than after 34 weeks. When the cutoff value was 97.2, the positive predictive value for diagnosing PE was as high as 97.5%. And the negative predictive value was 97.9% with a cutoff value of 21.5. For the sFlt-1/PlGF ratio between 21.5 and 97.2, we agreed with the other researchers to repeat the measurement in 1~2 weeks.

Although the number of GH patients was limited in our study, we can still find the trend in this group. About 50% (6/12) of GH cases had sFlt-1/PlGF ratios less than 21.5, 41.7% (5/12) were between 27.5 and 97.2. All of them had smooth pregnancy outcomes. Only one GH patient born a 1750 g baby at 35^+6^ weeks by C-section, who had a high sFlt-1/PlGF ratio of 207.7 at 34 weeks. After 34 weeks of gestation, the sFlt-1/PlGF ratio difference was not significant between PE and GH. This finding is in line with Noori et al. [38] but slightly different from those of von Dadelszen et al. [36] who found significant differences between GH and PE in both gestational weeks' groups. Our data also showed, when PE are ruled out, 86.7% (13/15) of chrHTN, 68.8% (11/16) of CTD, 84.6% (11/13) of proteinuria, and 98.7% (75/76) of normal controls had a sFlt-1/PlGF ratio less than 21.5. About 25% (11/44) of other medical complication had slightly higher ratio than 21.5 but none exceed 97.2; for these women, repeating the test in 1~2 weeks is also prudent.

Studies have shown that the risk, clinical manifestations, and severity of PE are related to the maternal race and ethnicity [39, 40]. For instance, compared to non-Hispanic white women, there was a slightly decreased risk for East Asian women and the highest risk for Mexican women. Those likely reflected a difference in the mechanism of hypertensive disease among different ethnic women. That was why cutoff values and predictive efficacy of the sFlt-1/PlGF ratio varied largely across study countries [23–26]. We conducted a study in Chinese women, clarified its value in diagnosis of PE and prediction of pregnancy duration, and proposed a cutoff value suitable for Chinese population first time. In addition, the very pregnancies with several internal medical complications were included in our study. Owing to the overlap about clinical manifestations, it is difficult to differentiate these diseases from PE sometimes. Our study indicated that the sFlt-1/PlGF ratio may be a workable serological marker for the differential diagnosis. At present, no similar reports have been found and the value deserves further study.

### 4.2. sFlt-1/PlGF Ratio as a Potential Indicator of Remaining Duration of Pregnancy in PE

Predicting the delivery time is crucial for timely administration of glucocorticoid for fetal lung maturation and transferring of the patients to the medical institution with NICU. Elevated blood pressure and proteinuria are the two most important clinical indexes in the diagnosis of PE, but they are far from satisfying to provide information about the clinical course of the disease or maternal and fetal adverse outcomes [41]. Verlohren [35] showed that the sFlt-1/PlGF ratio was correlated with the remaining pregnancy duration, thus allowing individualized risk stratification for an imminent delivery of PE patients. We supported their conclusion and found in our study that the sFlt-1/PlGF ratio in PE patients whose remaining pregnancy duration was within 7 d was significantly higher than those with more than 7 d especially in severe or early onset PE patients. All patients whose ratios were higher than 301.0 (Q3, the third quartile of the sFlt-1/PlGF ratio in early onset PE patients) were delivered within 7 d regardless of disease severity, while only 66.0% (33/50) of patients whose ratios were lower than 301.0 were delivered within 7 d (*P* = 0.018). These data have important clinical implication. It provides an independent classification method to the stratification of PE patients besides clinical criteria [42].

## 5. Conclusions

The serum sFlt-1/PlGF ratio is an efficient marker in the diagnosis and differential diagnosis of preeclampsia. This ratio can also be used to predict the timing of delivery for preeclampsia patients.

## Figures and Tables

**Table 1:** Baseline characteristics of study population.

	Controls	PE	GH	chrHTN	CTD	Proteinuria
Total (*n*)	76	62	12	15	16	13
Age (y)	29 ± 3	31 ± 5	33 ± 5	31 ± 5	31 ± 5	33 ± 4
Multipara (%)	18.4	28.6	16.6	13.3	0	16.7
Gestational week of test	30 ± 5	31 ± 4	33 ± 3	32 ± 4	32 ± 4	30 ± 4
Gestational week of delivery	39 ± 2	32 ± 4^a^	38 ± 2	39 ± 1	37 ± 3	39 ± 1
Birthweight (g)	3198 ± 388	1624 ± 703^a^	3130 ± 584	3167 ± 399	2639 ± 616	3080 ± 325
sFlt-1/PlGF ratio	4.1 ± 0.5	257.7 ± 44.7^a^	38.6 ± 17.4	8.9 ± 2.4	19.6 ± 6.9	10.5 ± 3.5

^a^Women with PE had a lower mean birthweight of the neonate, a shorter mean gestational week of delivery, and a higher sFlt-1/PlGF ratio than healthy controls or women with GH, chrHTN, CTD, or proteinuria. The difference was significant, *P* < 0.001.

**Table 2:** sFlt-1/PlGF ratio in PE, GH, chrHTN, and healthy controls.

Group	PE (*n* = 62)		Control (*n* = 76)		GH (*n* = 12)		chrHTN (*n* = 15)	
	<34 wks	≥34 wks	<34 wks	≥34 wks	<34 wks	≥34 wks	<34 wks	≥34 wks
Total (*n*)	48	14	59	17	5	7	10	5
sFlt-1/PlGF ratio	311.9 ± 55.4	71.7 ± 11.7	2.9 ± 0.2	8.3 ± 1.7	21.5 ± 18.8	50.8 ± 26.9	8.1 ± 3.0	10.4 ± 4.1
*P* value^a^	<0.001^a^		0.005^a^		0.432		0.665	

^a^The comparison of the sFlt-1/PlGF ratio between <34 weeks and ≥34weeks subgroups showed that PE patients <34 weeks had a significant higher average sFlt-1/PlGF ratio than patients ≥34 weeks; controls <34 weeks significantly decreased than controls ≥34 weeks.

## Data Availability

The data used to support the findings of this study are available from the corresponding author upon request.
